# Effects of аntimicrobials on Pseudomonas aeruginosa biofilm formation

**DOI:** 10.18699/VJGB-22-60

**Published:** 2022-08

**Authors:** U.M. Nemchenko, K.O. Sitnikova, N.L. Belkova, E.V. Grigorova, N.M. Voropaeva, M.V. Sukhоreva, E.S. Sukhareva, E.D. Savilov

**Affiliations:** Scientific Сentre for Family Health and Human Reproduction Problems, Irkutsk, Russia; Scientific Сentre for Family Health and Human Reproduction Problems, Irkutsk, Russia; Scientific Сentre for Family Health and Human Reproduction Problems, Irkutsk, Russia; Scientific Сentre for Family Health and Human Reproduction Problems, Irkutsk, Russia; Scientific Сentre for Family Health and Human Reproduction Problems, Irkutsk, Russia; City Ivano-Matreninskaya Children’s Clinical Hospital, Irkutsk, Russia; City Ivano-Matreninskaya Children’s Clinical Hospital, Irkutsk, Russia; Scientific Сentre for Family Health and Human Reproduction Problems, Irkutsk, Russia Irkutsk State Medical Academy of Postgraduate Education – Branch Campus of the Russian Medical Academy of Continuing Professional Education of the Ministry of Healthcare of the Russian Federation, Irkutsk, Russia

**Keywords:** Pseudomonas aeruginosa, biofilm formation, antimicrobial drugs, antibiotic resistance, Pseudomonas aeruginosa, биопленкообразование, антимикробные препараты, антибиотикорезистентность

## Abstract

Pseudomonas aeruginosa is one of the most problematic pathogens in medical institutions, which may be due to the ability of this microorganism to exist in a biofilm, which increases its resistance to antimicrobials, as well as its prevalence and survival ability in the external environment. This work aimed to evaluate the antimicrobial susceptibility of P. aeruginosa strains in planktonic and biofilm forms. We studied 20 strains of P. aeruginosa collected during 2018–2021 by specialists from the Laboratory of Microbiome and Microecology of the Scientific Centre for Family Health and Human Reproduction Problems. The identification of strains was carried out using test systems for differentiating gram-negative non-fermenting bacteria (NEFERMtest 24 Erba Lachema s.r.o., Czech Republic), and confirmed by mass spectrometric analysis and 16S rRNA gene sequencing. Antimicrobial activity was assessed by the degree of inhibition of cell growth in planktonic and biofilm forms (on a flat-bottomed 96-well plastic immunological plate). All clinical isolates of P. aeruginosa were biofilm formers, 47.6 % of the isolates were weak biofilm formers, and 52.4 % of the isolates were moderate biofilm formers. Planktonic cells and the forming biofilm of the tested P. aeruginosa strains were carbapenems-resistant. Biofilm formation was suppressed in more than 90 % of cases by the agents of the cephalosporin and aminoglycoside groups. Antimicrobial susceptibility of P. aeruginosa strains in the formed biofilm was significantly lower (p < 0.05). Carbapenems and cephalosporins did not affect the mature biofilms of the tested P. aeruginosa strains in more than 60 % of cases. Only non-beta-lactam antibiotics (ciprofloxacin and amikacin) suppressed the growth of planktonic cells and destroyed the mature biofilm. The revealed differences in the effect of the tested antimicrobials on the P. aeruginosa strains biofilms correlate with resistance to a number of antibiotics. To prevent biofilm formation in the hospital strains of P. aeruginosa, the use of ceftazidime may be recommended, and antimicrobials such as ciprofloxacin and amikacin may be used to affect mature biofilms of P. aeruginosa.

## Abstract (ru)

Синегнойная палочка (Pseudomonas aeruginosa) относится к наиболее проблемным патогенам в лечебных учреждениях, что может быть связано со способностью этого микроорганизма существовать в биопленке, которая повышает его устойчивость к антимикробным препаратам, а также распространенность и выживаемость во внешней среде. Цель настоящей работы – оценка чувствительности штаммов P. aeruginosa, находящихся в планктонной форме и форме биопленки, к воздействию антимикробных препаратов. Исследовано 20 штаммов P. aeruginosa из рабочей коллекции лаборатории микробиома и микроэкологии Научного fluoroquinoцентра проблем здоровья семьи и репродукции человека, собранной в течение 2018–2021 гг. Идентификация штаммов проведена с использованием тест-систем для дифференциации грамотрицательных неферментирующих бактерий и подтверждена масс-спектрометрическим анализом и секвенированием гена 16S рРНК. Ак- тивность антимикробных препаратов оценивали по степени ингибирования роста клеток бактерий, находя- щихся в планктонной форме и форме биопленки. Установлено, что все клинические штаммы P. aeruginosa были биопленкообразующими, 47.6 % относились к слабообразующим, 52.4 % – к умереннообразующим. Планктон- ные клетки и формирующаяся биопленка тестируемых штаммов были устойчивы к карбапенемам. Формиро- вание биопленки в более чем 90 % случаев подавляло препараты групп цефалоспоринов и аминогликозидов. Чувствительность к воздействию антимикробных препаратов у штаммов P. aeruginosa, находящихся в сфор- мированной биопленке, была значимо ниже ( p < 0.05). Карбапенемы и цефалоспорины не воздействовали на зрелые биопленки тестируемых штаммов P. aeruginosa более чем в 60 % случаев. Только не-бета-лактамные антибиотики (ципрофлоксацин и амикацин) подавляли рост планктонных клеток и разрушали зрелую био- пленку. Выявленные различия в действии испытанных препаратов на биопленку штаммов P. aeruginоsa кор- релируют с устойчивостью к целому ряду антибиотиков. Для предупреждения формирования биопленок у больничных штаммов P. аeruginosa может быть рекомендовано применение цефтазидима, для воздействия на зрелые биопленки P. аeruginosa – антимикробные препараты ципрофлоксацин и амикацин.

## Introduction

Pseudomonas aeruginosa invariably occupies the leading place among pathogens of nosocomial infections in the Russian Federation and is included in the group of opportunistic bacteria, united by the term ESKAPE (Skleenova et al., 2018). The presence of a wide range of pathogenic factors, genetic flexibility, and the ability to rapidly acquire resistance to different antibiotic groups makes P. aeruginosa one of the most problematic pathogens in healthcare settings (Edelstein et al., 2019). Patients with compromised immune systems, eye burns and trauma, and those with internal medical devices are primarily at risk of developing a pseudomonal infection (Diggle, Whiteley, 2020). Pseudomonal infections are particularly dangerous in patients with cystic fibrosis (Kosztołowicz et al., 2020; Scherz et al., 2021).

Treatment of infections caused by P. aeruginosa is complicated by the ability of these bacteria to exist in a biofilm, which increases their resistance to antibiotics, their prevalence, and survival ability (de Abreu et al., 2014; Olivares et al., 2020). Destruction of bacterial biofilms formed in the secretions of cystic fibrosis patients was shown to be a serious problem, since diffusion of antibiotics into biofilm structures is poor, and their antibacterial activity can stimulate drug resistance (Kosztołowicz et al., 2020). Classical methods for determining antibiotic sensitivity (broth or agar dilution methods and disc diffusion method) are performed on non-adherent bacteria. The results obtained with these methods cannot predict the therapeutic success of the respective antibiotics against biofilms (Olivares et al., 2020). Currently, there are no guidelines to help clinicians treat biofilm infections, which gives reason for developing routine laboratory methods to determine the sensitivity of biofilm bacteria to antibiotics (Olivares et al., 2020).

According to the experts of the European Committee on Antimicrobial Susceptibility Testing (EUCAST), the variety of antipseudomonal antibiotics, sensitivity to which is evaluated under in vitro conditions, includes penicillins, cephalosporins, carbapenems, monobactams, fluoroquinolones, aminoglycosides and polymyxins1.European Committee on Antimicrobial Susceptibility Testing [electronic source]. Clinical breakpoints – breakpoints and guidance. URL: http://www.eucast.org/clinical_breakpoints/ (accessed on: 15 October 2021). In this regard, we studied the effect of the above groups of antimicrobial agents (AMAs) (ceftazidime, cefepime, imipenem, meropenem, ciprofloxacin and amikacin) on plankton cell growth, forming and mature P. aeruginosa biofilm.

The aim of the study was to evaluate the sensitivity of P. aeruginosa strains in the planktonic form and in the biofilm form to antimicrobial agents.

## Materials and methods

The objects of the study were 20 strains of P. aeruginosa with confirmed drug resistance to antimicrobials from the collection of the Laboratory of Microbiome and Microecology of the Scientific Centre for Family Health and Human Reproduction Problems, accumulated during 2018–2021. Type strain P. aeruginosa ATCC 27853 (Scientific Centre “Kurchatov Institute” – Research Institute for Genetics and Selection of Industrial Microorganisms) was used as a control.

Hospital strains were isolated from patients from two medical institutions in Irkutsk according to the principle “one patient–one isolate”. Eight cultures were obtained from the Irkutsk State Regional Children’s Clinical Hospital (Noskova et al., 2020) and 12 cultures were obtained from the City Ivano-Matreninsky Children’s Clinical Hospital. Cultures were gathered from patients with different types of diseases (sepsis, acute hematogenous osteomyelitis, peritonitis, pneumonia, etc.) and isolated from oropharynx, liquor, wound, endotracheal tubes, tracheostomy, central venous catheter (14 cultures). A separate group consisted of 6 cultures isolated from the sputum of patients with such a genetic disease as cystic fibrosis (CF).

Identification of P. aeruginosa strains. Primary differentiation of P. aeruginosa strains was performed by colony morphology, pigment on blood agar, and Gram staining. Biochemical identification of selected cultures was performed using test systems for differentiation of Gram-negative nonfermenting bacteria NEFERMtest 24 (Erba Lachema s.r.o., Czech Republic), and confirmed by MALDI-TOF using direct protein profiling of nonfermenting microorganisms. Mass spectrometric analysis was performed on the Bruker UltrafleXtreme mass spectrometer (Bruker Daltonics, Germany). Additionally, cultures were identified by a fragment of the ribosomal operon containing the V1–V4 variable regions of the 16S rRNA gene. Full-length 16S rRNA gene fragments of P. aeruginosa strains were registered in the international GenBank database under numbers OL616031–OL616034.

To assess the effect of AMA on biofilm formation and destruction of the formed biofilms, antibiotics of the following groups were used: cephalosporins, carbapenems, aminoglycosides, fluoroquinolones, in the form of standard cardboard disks with antimicrobial drugs DI-PLS-50-01, (NICP, Research Centre for Pharmacotherapy, Russia), HiMedia Laboratories Pvt. Limited (India).

Determination of biofilm formation capacity and biofilm resistance to AMAs using 96-well plastic plates. A 24-hour culture was used for the assay. The inoculum was densified in meat-peptone broth (MPB) to 106 CFU/mL. Strains were prepared, culture optical density (OD) was measured, biofilms were stained, and the biofilm formation intensity was determined by measuring the optical density with gentian violet/ ethanol extracts, and the biofilm formation coefficient (BFC) was calculated according to the previously described methods (Nemchenko et al., 2020; Grigorova et al., 2021).

Evaluation of the ability of AMA to affect plankton cell growth and biofilm formation. To determine the ability of AMAs to affect plankton cells and the forming biofilm, one AMA disk with the required antibiotic concentration was added to the plate simultaneously with a 24-hour culture: ceftazidime – 10 μg, cefepime – 30 μg, imipenem – 10 μg, meropenem – 10 μg, ciprofloxacin – 5 μg, amikacin – 30 μg. Sterile MPB served as a control. After 30 min, the disks were removed (Tapalskiy, Bilskiy, 2018), the plates were cultured in the thermostat for 24 h, then the experiments were conducted as previously described (Nemchenko et al., 2020; Grigorova et al., 2021).

Evaluation of the ability of AMA to destroy mature biofilms. To determine the ability of AMA to destroy a mature biofilm, plankton cells were removed from the culture plate after 24 h of incubation, washed three times with sterile distilled water, and 150 μL of sterile MPB and one AMA disk were added to each well, including control wells. The disks were removed after 30 min. The plates were incubated for another 24 h. Furthermore, the procedure was similar to that previously described (Nemchenko et al., 2020; Grigorova et al., 2021).

Registration of experimental results. The biofilm formation coefficient (BFC) was calculated after measuring the optical density of the ethanol extract of the stained wells in all plates as the ratio of the optical density of the experiment extract and optical density of the control extract. When the obtained BFC values were less than 2.0, strains were classified as weak biofilm formers, with values of 2.0–3.9, as moderate biofilm formers, and above 3.9, as strong biofilm formers (Nemchenko et al., 2020; Grigorova et al., 2021). The effect coefficient of AMA on forming and mature biofilms was calculated using the formula OD BFform/OD BFwithout AMA or OD BFmature/OD BFwithout AMA, where OD BFform or OD BFmature is the optical density of the ethanol extract of the biofilm influenced by AMA, OD BFwithout AMA is the optical density of the ethanol extract of biofilm cultures without the AMA effect. With a ratio < 0.9, AMA was considered to affect the biofilm; from 0.9 to 1.0, AMA had little effect on the biofilm; from 1.0 and above, AMA had no effect on the biofilm.

The growth of plankton cells in the plate wells was determined as the ratio of the optical density of the bacterial plankton cell suspension after 24 h of cultivation to the initial density; the result was interpreted as previously described (Nemchenko et al., 2020; Grigorova et al., 2021).

Statistical processing of the data was performed using licensed MS Excel 2007 for Windows 7 applications. Nonparametric criteria were used to assess the significance of differences between the two groups according to the level of any criterion: χ2, Mann–Whitney U-criterion. Absolute and relative (percentage) values were calculated for the qualitative variables. The significance level for statistical hypothesis testing (p) was assumed to be 0.05.

## Results

It was found that under laboratory conditions without AMA exposure, the planktonic cells of P. aeruginosa had a significant growth rate (Table 1). The density of microbial cells increased in 24 h of cultivation more than ten-fold compared to the initial density (Uemp = 0, differences significant between the initial density and the density after 24 h, Mann–Whitney test).

**Table 1.**
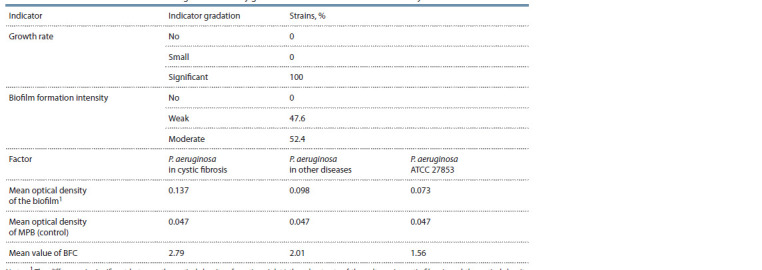
Characterization of the tested P. aeruginosa strains by growth rate and biofilm formation intensity The difference is significant between the optical density of gentian violet/ethanol extracts of the cultures in cystic fibrosis and the optical density of gentian violet/ethanol extracts of P. aeruginosa ATCC 27853, Uemp = 1 Mann–Whitney test, p < 0.01. MPB – meat-peptone broth; BFC – biofilm formation coefficient.

The OD of P. aeruginosa biofilm cultures isolated from sputum in such a severe, genetically determined disease as cystic fibrosis was significantly greater than that of the type strain (p < 0.01) and cultures isolated in other diseases (see the Figure). A similar pattern was observed when comparing BFCs. The mean BFC of cystic fibrosis P. aeruginosa was 2.79 ± 0.78; P. aeruginosa in other diseases was 2.01 ± 0.69; P. aeruginosa ATCC 27853 was 1.56.

**Fig. 1.**
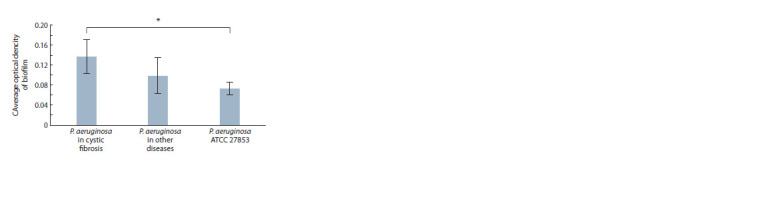
Mean value of biofilm optical density of the tested P. aeruginosa strains. The difference is significant between the optical density of biofilm cultures in cystic fibrosis and the optical density of biofilm of P. aeruginosa ATCC 27853, Uemp=1 Mann–Whitney test, p < 0.01.

Evaluation of biofilm formation ability by the amount of dye bound to the biofilm showed that the strains studied, including the P. aeruginosa ATCC 27853 type strain, were weak biofilm formers in 47.6 %, in 52.4 % of cases were moderate biofilm formers (see Table 1).

A comparison of the optical densities of cultures growing without and under the AMA effect showed that planktonic cells were resistant to AMA imipenem (5 % of sensitive cultures, Uemp = 46) and meropenem (5 % of sensitive cultures, Uemp = 64.5; there is a difference between the initial density and the density after 24 h, Mann–Whitney test, p < 0.05). The other drugs inhibited the growth of planktonic cells, the most effective were amikacin (60 % of sensitive cultures, Uemp = 180.5) and ciprofloxacin (50 % of sensitive cultures, Uemp = 191.5), cefepime affected 40.0 % of cultures (Uemp = 191.5), and ceftazidime suppressed the growth of P. aeruginosa cultures in 35 % of cases (Uemp = 179.0) (no difference between the initial density and the density after 24 h, Mann–Whitney test, p > 0.05).

Study of the ability of AMAs to affect the formation and destruction of mature biofilms

The ability of AMAs to affect biofilm formation in P. aeruginosa cultures was evaluated using the ratio of the optical density of biofilms exposed to AMAs to the optical density of biofilms without AMA exposure.

The studies showed that not all AMAs prevent biofilm formation (Table 2). Ciprofloxacin had no effect on biofilm formation in 23.8 % of cases, imipenem and meropenem, in 33.3 and 38.1 %, respectively; ceftazidime, cefepime, and amikacin were most effective in suppressing biofilm formation. Significant differences were found only for ceftazidime, which most effectively suppressed biofilm formation, compared with imipenem (χ2 = 5.62) and meropenem (χ2 = 7.03) (p < 0.05).

**Table 2.**
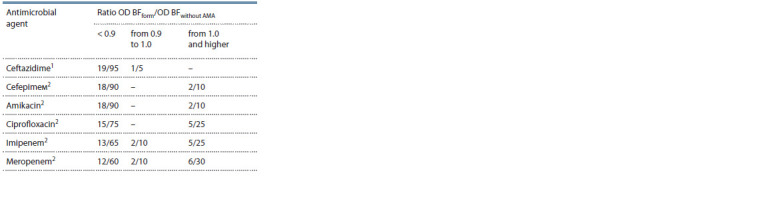
Ability of AMAs to affect biofilm formation of the tested P. aeruginosa strains (absolute value/%) Ceftazidime affects biofilm formation compared to imipenem and meropenem, p < 0.05; 2 no difference when comparing the effect between other AMAs, p > 0.05; OD BFform – optical density of the forming biofilm under the effect of AMA; OD BFwithout AMA – optical density without AMA exposure; AMAs – antimicrobial agents.

The sensitivity of P. aeruginosa cells in a mature biofilm to AMA exposure was lower than that of biofilm formation (Mann–Whitney test, difference significant between the optical density of a forming biofilm and a mature biofilm, p < 0.05). AMAs ceftazidime, cefepime, imipenem, and meropenem had little or no effect on P. aeruginosa biofilms; the BFmature/BFwithout AMA ratio was 0.9 or higher in more than 60 % of cases. Only non-beta-lactam antibiotics, such as amikacin and ciprofloxacin, affected the formed biofilm (Table 3). Comparison of the AMAs effects among themselves showed that amikacin was more effective than ceftazidime (χ2 = 5.01) and meropenem (χ2 = 10.98), ciprofloxacin was more effective than meropenem (χ2 = 7.62).

**Table 3.**
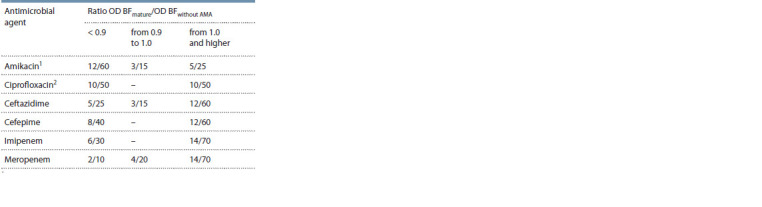
Ability of different AMAs to affect the mature biofilm of P. aeruginosa strains (absolute value/%) Amikacin destroys the mature biofilm compared with ceftazidime (p = 0.02) and meropenem (p <0.001); 2 ciprofloxacin destroys the mature biofilm compared to meropenem (p< 0.05); OD BFmature – optical density of the mature biofilm under the AMA effect; OD BFwithout AMA – optical density of the biofilm with no AMA effect; AMAs – antimicrobial agents.

The BFC of P. aeruginosa strains in the formed biofilm was significantly higher than BFC of cultures exposed to AMAs at the stage of biofilm formation, which also confirms the resistance of the mature biofilm. BFC for ceftazidimeform/mature Uemp = 48.5; cefepimeform/mature Uemp = 58; imipenemform/mature Uemp = 97; amikacinform/mature Uemp = 50. There is a difference between the BFC value of the forming and BFC value of the mature biofilm, Mann–Whitney test, p <0.01.

## Discussion

The experiment showed that not all AMAs inhibited the growth of planktonic cells of clinical P. aeruginosa isolates. Resistance to cephalosporins (ceftazidime and cefepime) was demonstrated by 65 and 60 % of the tested strains, respectively. Resistance to carbapenems (imipenem and meropenem) was observed in almost all isolates. Resistance to non-beta-lactam antibiotics (amikacin and ciprofloxacin) was shown by 40 and 50 % of the strains, respectively. The findings are consistent both with our previous studies (Noskova et al., 2020) and with a multicentre epidemiological study of antibiotic resistance of nosocomial pathogens (“MARATHON” 2015–2016), which observed an increase in resistance of nosocomial P. aeruginosa strains to most AMAs, including carbapenems (Edelstein et al., 2019).

The strains studied, especially those isolated from patients with cystic fibrosis, were biofilm-forming (see Table 1). This served as the basis for us to evaluate the effectiveness of AMAs against the forming biofilm of nosocomial pathogens. The experiment showed that compared to other antibiotics, ceftazidime was the most effective drug inhibiting biofilm formation (see Table 2).

As recent studies show, in addition to classical resistance mechanisms, bacteria are able to withstand exposure to high antibiotic concentrations by exhibiting so-called tolerance (Brauner et al., 2016; Yan, Bassler, 2019). Tolerant bacteria grow more slowly than their non-tolerant counterparts and may avoid death by antibiotic treatment (Brauner et al., 2016). Another form of tolerance, which does not result from inherited mutations but rather from phenotypic differentiation, is commonly referred to as persistence. Time-dependent destruction of the bacterial population by antibiotics shows that actively growing cells die first, while persistent cells die in the second phase at a much lower rate. It is this subset of microorganisms that survives antibiotic exposure and recovers after antibiotic withdrawal (Balaban et al., 2004).

It has been suggested that the ability of biofilms to contain tolerant and persistent cells underlies the difficulties encountered in eliminating biofilms (Lewis, 2012). It is likely that the increased antibiotic tolerance arises from altered biofilm cell physiology. It has been suggested that cells within biofilms are in a stationary phase where the penetration of nutrients and oxygen is limited due to consumption by the cells located peripherally (Yan, Bassler, 2019). The presence of persistent cells can be dangerous in certain groups of patients, such as those with cystic fibrosis, when highly persistent mutants are released after long-term antibiotic treatment (Lewis, 2012).

The studies presented showed that the sensitivity of cells in mature biofilms to AMAs was significantly lower; the antibiotics generally failed to destroy biofilm cultures of P. aeruginosa. The BFC of cultures in mature biofilms was higher than that of cultures that were affected by AMA during biofilm formation (p < 0.01).

Of all AMAs tested, only non-beta-lactam antibiotics (ciprofloxacin and amikacin) inhibited the growth of plankton cells and destroyed the mature biofilm, which may be related to the mechanism of the effect of different classes of antibiotics. The cells in the biofilm decrease the rate of cell division, making them less sensitive to beta-lactam antibiotics affecting the cell wall, while the effect of ciprofloxacin and amikacin does not require actively dividing cells since it targets transcription and translational processes (Sidorenko et al., 2013; Thieme et al., 2021).

The most effective approach to prevent biofilm formation would be to inhibit the adhesive capacity of cells (Olivares et al., 2020). For example, a study by S. Otani et al. (2018) showed that subinhibitory minimal suppressive concentrations of ceftazidime reduced biofilm mass, suppressed motility and expression of genes involved in bacterial adhesion and P. aeruginosa PAO1 matrix production (Otani et al., 2018). Previously, S. Roudashti et al. (2017) observed the effects of cephalosporins in P. aeruginosa QS systems providing motility and biofilm formation in these microorganisms (Roudashti et al., 2017). In our study, ceftazidime also showed the highest antibiofilm effect compared with other AMAs. However, the mechanism of biofilm resistance to AMAs is complex, multifactorial, and contradictory. This point is supported by numerous studies that demonstrate that low doses of antimicrobials in the centre of infection can increase the risk of mutagenesis and initiate biofilm formation (Kaplan, 2011; Ciofu et al., 2015; Olivares et al., 2020).

## Conclusion

Thus, the study of the effect of AMAs of the groups of cephalosporins, carbapenems, fluoroquinolones and aminoglycosides on the biofilms of the tested hospital P. aeruginosa strains showed that the antipseudomonal drugs mainly prevented the formation but did not destroy the already formed biofilm. The significant differences detected in the effect of the tested AMAs both on the mature biofilm of P. aeruginosa strains and on the process of its formation to a certain extent correlate with the resistance of this microorganism to a number of antibiotics (Edelstein et al., 2019; Adzhieva et al., 2021). Additional research aimed at detecting tolerant and persistent cells is needed to elucidate the mechanisms involved, which will optimise the overall use of antimicrobials for treating biofilm-related infections (Yan, Bassler, 2019). The use of ceftazidime may be recommended to prevent biofilm formation in the hospital strains of P. aeruginosa, and amikacin and ciprofloxacin may be recommended for affecting mature P. aeruginosa biofilms.

## Conflict of interest

The authors declare no conflict of interest.
